# Comparison of ^99m^Tc-Tetrofosmin and ^99m^Tc-Sestamibi Uptake in Glioma Cell Lines: The Role of P-Glycoprotein Expression

**DOI:** 10.1155/2014/471032

**Published:** 2014-11-10

**Authors:** George A. Alexiou, Xanthi Xourgia, Evrysthenis Vartholomatos, Spyridon Tsiouris, John A. Kalef-Ezra, Andreas D. Fotopoulos, Athanasios P. Kyritsis

**Affiliations:** ^1^Neurosurgical Institute, University of Ioannina, Stavros Niarchos Avenue, 45500 Ioannina, Greece; ^2^Department of Nuclear Medicine, University Hospital of Ioannina, Stavros Niarchos Avenue, 45500 Ioannina, Greece; ^3^Department of Medical Physics, University of Ioannina, Stavros Niarchos Avenue, 45500 Ioannina, Greece

## Abstract

^99m^Tc-Tetrofosmin (^99m^Tc-TF) and ^99m^Tc-Sestamibi (^99m^Tc-MIBI) are SPECT tracers that have been used for brain tumor imaging. Tumor's multidrug resistance phenotype, namely, P-glycoprotein (p-gp), and the multidrug resistance related proteins (MRPs) expression have been suggested to influence both tracers' uptake. In the present study we set out to compare ^99m^Tc-TF and ^99m^Tc-MIBI uptake in high-grade glioma cell lines and to investigate the influence of gliomas p-gp expression on both tracers' uptake. We used four glioma cell lines (U251MG, A172, U87MG, and T98G). The expression of p-gp protein was evaluated by flow cytometry. Twenty *μ*Ci (7.4*·*10^5^ Bq) of ^99m^Tc-TF and ^99m^Tc-MIBI were used. The radioactivity in the cellular lysate was measured with a dose calibrator. P-gp was significantly expressed only in the U251MG cell line (*P* < 0.001). In all gliomas cell lines (U251MG, U87MG, A172, and T98G) the ^99m^Tc-TF uptake was significantly higher than ^99m^Tc-sestamibi. The U251MG cell line, in which significant p-gp expression was documented, exhibited the strongest uptake difference. ^99m^Tc-TF uptake was higher than ^99m^Tc-MIBI in all studied high-grade glioma cell lines. Thus, ^99m^Tc-TF may be superior to ^99m^Tc-MIBI for glioma imaging *in vivo*.

## 1. Introduction

^99m^Tc-Tetrofosmin (^99m^Tc-TF) and ^99m^Tc-sestamibi (^99m^Tc-MIBI) are tracers that have been used among others for brain tumor imaging [1, 2]. Both agents have been used for the differentiation of glioma recurrence from treatment induced necrosis [3, 4], neoplastic from nonneoplastic intracerebral hemorrhage [5], assessment of glioma and meningioma aggressiveness and patient's prognosis [6, 7]. Nevertheless, several studies proposed that ^99m^Tc-TF might be superior to ^99m^Tc-MIBI for brain tumor imaging, since the latter is influenced to a greater degree by tumor's multidrug resistance phenotype (MDR) [8–10]. MDR phenotype is associated with P-glycoprotein (p-gp) and multidrug resistance related proteins (MRPs) expression. These proteins act as membrane-efflux transporters that pump drugs out of the cancer cells and reduce intracellular concentration [11]. Both ^99m^Tc-TF and ^99m^Tc-MIBI are substrates of these pumps [8, 9]. In the present study we set out to compare ^99m^Tc-TF and ^99m^Tc-MIBI uptake in four high-grade glioma cell lines and the influence of p-gp expression on tracer uptake.

## 2. Material and Methods

### 2.1. Cell Lines

The human glioma cell lines U251MG and A172 were obtained from Dr. W. K. Alfred Yung (Department of Neuro-Oncology, M.D. Anderson Cancer Center, Houston, TX). U87MG and T98G were obtained from American Type Culture Collection (ATCC; Manassas, VA, USA). They were cultured in Dulbecco's modified Eagle's medium (DMEM, Gibco BRL, Life Technologies, Grand Island, NY) supplemented with 10% fetal bovine serum (FBS) and 1% penicillin/streptomycin (Gibco, BRL) and grown at 37°C in a 5% CO_2_ atmosphere as described in detail elsewhere [12].

### 2.2. P-Glycoprotein Staining and Detection

P-gp staining was accomplished using a PE-conjugated monoclonal antibody against human p-gp (Anti-P-glycoprotein clone 15D3, Becton Dickinson Immunocytometry Systems, San Jose, USA) in accordance with standard surface staining protocols. In brief, cells were stained for 30 min in the dark at room temperature (20° to 25°C). After completion of staining, cells were washed in PBS and resuspended in 500 *μ*L PBS. Cells were kept on ice until being analyzed by flow cytometry within 1 h. Antibody specificity was controlled by staining of all cell lines with an isotype-matched control antibody (IgG1-PE, isotype control; Becton Dickinson Immunocytometry Systems, San Jose, USA). Flow cytometric analysis of p-gp stained cells was performed on a FACSCalibur (BD Bioscience) equipped with a standard argon laser for 488 nm excitation and with 530/30 nm band pass (FL1), 585/42 nm band pass (FL2), and 670 long pass (FL3) filters. Results were evaluated using CellQuest software (Becton Dickinson, San Jose, USA).

### 2.3. Radioactive Tracer Experiments

^99m^Tc-Tetrofosmin (Myoview, GE Healthcare, UK) and ^99m^Tc-sestamibi (Stamicis, Cis bio International) were prepared according to the manufacturer instructions. The radiochemical purity of each radiotracer was greater than 95%.

### 2.4. Preliminary Experiments

**Figure 1:**
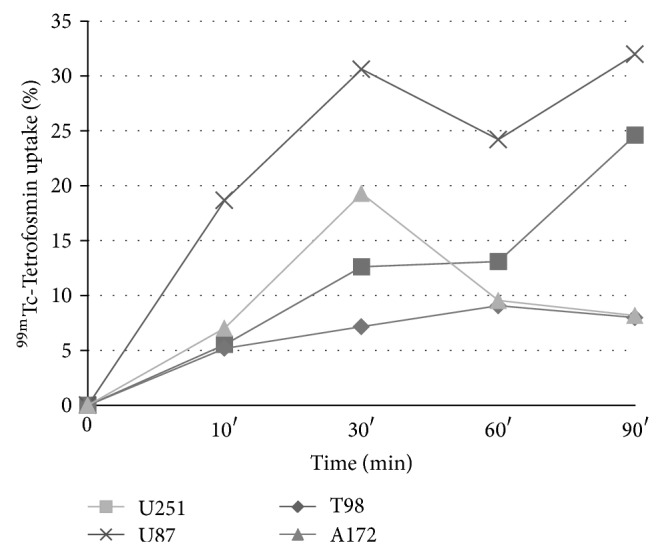
Uptake (%) of ^99m^Tc-TF per cell type. Tracer uptake was practically linear for incubation times up to 30 min in all cell lines (lines were drawn to facilitate the eye).

Initially, time activity curves for all cell lines were constructed to study the optimum time for the calculation of tracer activity. More specifically, about 5 × 10^3^ cells were platted per each 4 cm plate. At the fourth day 10 *μ*Ci (3.7*·*10^5^ Bq) (200 *μ*L) of ^99m^Tc-Tetrofosmin was added to the medium. We used four time points (10, 30, 60, and 90 min) for incubation with the tracer and then the medium was discarded. The cells were rapidly washed three times with phosphate buffered saline (PBS) at 4°C. Cells were then treated with 0.5 mL of trypsin. When the cells had detached from the bottom of the well (within 5 min), 1 mL of DMEM was added to stop the proteolytic action. Cell clumps were removed by repeated (at least 10-fold) pipetting of the trypsin/DMEM mixture. The cells were then harvested. The radioactivity in the cellular lysate was counted ten times with a gamma scintillation counter (Wizard 2, Perkin Elmer, USA). Tracer's uptake was increased practically linearly with incubation times up to 30 min. Previous studies on ^99m^Tc-MIBI have used an incubation period of 30 min [13, 14]. Therefore, an incubation time of 30 min was chosen in all experiments (Figure 1).

### 2.5. Cell Kinetic Studies

About 5 × 10^5^ cells were platted per each 10 cm plate. At the fourth day 20 *μ*Ci (7.4*·*10^5^ Bq) (200 *μ*L) of each tracer was added to the medium. After 30 min of incubation with each tracer, the medium was discarded. The cells were scraped from the dishes. The radioactivity in the cellular lysate was counted with a dose calibrator (VDC 550, Veenstra, The Netherlands) found to have linear response down to 15 kBq of technetium-99 (^99m^Tc), the lowest studied activity The results were expressed as the percentage of the administered activity. All experiments were performed in duplicate and repeated three times.

## 3. Statistical Analysis

Unless otherwise stated, data are expressed as mean ± SD. The significance of differences between experimental conditions was determined using Mann-Whitney test. The Kolmogorov-Smirnov (KS) statistic, expressed as a *D* value, was used to compare binding of antibodies and of matched isotype controls. Differences were considered significant at *P* values less than 0.05.

## 4. Results

### 4.1. Study of the P-gp in Glioma Cells

**Figure 2:**
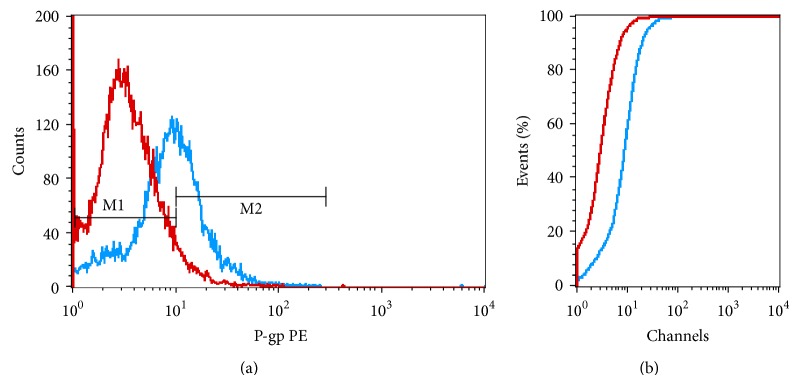
Illustration of flow cytometric and KS aspects. (a) Overlay histogram showing cell-surface P-gp protein expression in U251MG (blue line). The negative control antibody (red line) was mouse IgG1. (b) P-gp protein expression was, also, analyzed using the Kolmogorov-Smirnov (KS) statistic test (*D* value), which allows the objective and accurate identification of small differences in fluorescence intensity. Samples were considered positive when *D* ≥ 0.15.

P-gp was significantly expressed in the U251MG cell lines (*P* < 0.001) (Figure 2). On the contrary, no statistically significant p-gp expression was found in A172, U87MG and T98G cell lines.

### 4.2. ******^99m^Tc******-Tetrofosmin versus******^99m^Tc******-Sestamibi

The ^99m^Tc-TF uptake, ranging between ~21% and 22% in the four studied cell lines (Table 1), was higher than ^99m^Tc-MIBI in the four studied glioma cell lines (16% to 18%). In U251MG glioma cell the percentage of ^99m^Tc-Tetrofosmin uptake was significantly higher than that of ^99m^Tc-sestamibi (21.0 ± 0.4% versus 16.7 ± 0.9%,  *P* < 0.0001). In U87MG there was also significant higher ^99m^Tc-Tetrofosmin uptake (22.15 ± 1% versus 16.1 ± 1.9%,  *P* = 0.002). In A172 cell line the difference was also statistically significant (21.4 ± 1.3% versus 18.25 ± 0.8%,  *P* = 0.0017). In T98G there was higher ^99m^Tc-Tetrofosmin uptake compared to ^99m^Tc-sestamibi and the difference was also statistically significant (22.1 ± 1.6% versus 17.6 ± 0.95%,  *P* = 0.0002).

## 5. Discussion

The present study compared the uptake in four high-grade glioma cell lines of the monovalent lipophilic cationic diphosphine TF labeled with ^99m^Tc to that of ^99m^Tc-MIBI. The results showed higher ^99m^Tc-TF uptake, thus suggesting that ^99m^Tc-TF could be superior to ^99m^Tc-MIBI for glioma imaging. Apart from U251MG cell line, in which significant p-gp expression was documented, significant uptake difference was found in the other cell lines suggesting that other mechanisms may be implicated apart from p-gp expression.

^99m^Tc labeled compounds have been proven advantageous in tumor imaging over ^201^Tl due to higher number of photons of appropriate energy emitted by the human body per administered activity and lower radiation burden to both patient and members of the general public [15]. Regarding the mechanism of tracer uptake, ^99m^Tc-MIBI diffuses passively through the cell membrane and an estimated 95% of intracellular ^99m^Tc-MIBI is localized in mitochondria because of the negatively charged mitochondrial membrane. ^99m^Tc-TF enters viable cells mainly via passive transport, driven by the negative electric potential of the intact cell membrane, and it mostly localizes within the cytosol, with only a small fraction passing into the mitochondria [15].

Chemoresistance is a major obstacle for effective cancer treatment and can be present in a tumor at the time of initial diagnosis or can develop following treatment with chemotherapeutic agents [16]. One mechanism involved is the presence of a multidrug resistance phenotype from the tumor cells. Various genes have been implicated such as* MDR1*,* MRPs*, major vault protein (*MVP*) gene, the* MGMT* gene, and the* Survivin* gene [17]. The previous genes can produce resistance to a diverse range of drugs such as vincristine, temozolomide, etoposide, and cisplatin, whereas certain radiotracers are also substrate [11, 16, 17]. The* MDR1* gene is the most extensively studied [18]. This gene encodes a transmembrane p-glycoprotein that produces a broad pattern of resistance to several structurally and functionally unrelated drugs by expelling them out of the cells. Consequently, it reduces the intracellular drug concentration. P-gp is an important functional component of the blood-brain barrier [18]. MRPs are members of the ABC superfamily of transmembrane proteins that act as ATP-dependent drug efflux pumps and so far nine MRP members have been identified [3]. MRPs have been reported to confer resistance to various anticancer drugs and, in gliomas, MRP1, MRP3, MRP4, and MRP5 have been reported to be expressed more than the other members [19, 20].

Perek et al. studied the effect of glutathione (GSH) depletion on the chemosensitivity of human malignant glioma cell lines [21]. None of the glioma cell lines used by the authors were p-gp positives but were found to overexpress the MRP1 protein. In glioma cells, the multidrug resistance proteins are usually involved rather than P-gp, which is usually expressed in vessels. The authors found that both Tetrofosmin and MIBI are substrates of p-gp and MRP1; however both tracers did not follow the expected behavior of a MDR in all cases suggesting the presence of other mechanisms. In addition, ^99m^Tc-TF was more lipophilic than MIBI and thus could enter easier than MIBI in glioma cells [21].

In the present study U251MG cell line exhibited increased p-gp expression, in accordance with published data [22]. Similar to Perek et al. [21], we believe that, apart from p-gp, other mechanisms may also exist that influence tracer uptake. These mechanisms might explain the difference in tracer uptake in the remaining glioma cell lines that did not exhibit significant p-gp expression. In a previous study we investigated the MRP5 expression which can be normally found in the astrocytes of the subcortical white matter and in the pyramidal neurons [10]. In glioma patients we found that ^99m^Tc-TF uptake was not related to the MRP5 immunohistochemical expression; consequently, this might be another reason explaining the higher ^99m^Tc-TF uptake in the studied glioma cell lines.

A limitation of the present study was the absence of positive and negative control cells lines in order to define MDR protein expression. Thus, we cannot rule out the existence of an experimental bias regarding the uptake of both radiotracers that could influence their relationship with P-gp protein expression. In conclusion, the present study showed that ^99m^Tc-TF uptake is higher than that of ^99m^Tc-MIBI in all high-grade glioma cell lines studied. Thus, ^99m^Tc-TF is anticipated to be a superior tracer to ^99m^Tc-MIBI for gliomas imaging* in vivo*. Further studies are needed in order to elucidate the exact mechanisms involved in ^99m^Tc-TF uptake.

## Figures and Tables

**Table 1:** Tracer uptake 30 min postincubation in four glioma cell lines.

Cell line	^ 99m^Tc-Tetrofosmin	^ 99m^Tc-Sestamibi	*P* value
A172	21.4 ± 1.3%	18.25 ± 0.8%	0.0017
U87MG	22.15 ± 1.0%	16.1 ± 1.9%	0.002
U251MG	21.0 ± 0.4%	16.7 ± 0.9%	<0.0001
T98G	22.1 ± 1.6%	17.6 ± 0.95%	0.0002
